# High expression of AKR1B10 predicts low risk of early tumor recurrence in patients with hepatitis B virus-related hepatocellular carcinoma

**DOI:** 10.1038/srep42199

**Published:** 2017-02-09

**Authors:** Yan-Yan Wang, Lu-Nan Qi, Jian-Hong Zhong, Hong-Gui Qin, Jia-Zhou Ye, Shi-Dong Lu, Liang Ma, Bang-De Xiang, Le-Qun Li, Xue-Mei You

**Affiliations:** 1 Department of Hepatobiliary Surgery, Affiliated Tumor Hospital of Guangxi Medical University, Nanning 530021, PR China; 2 Key Laboratory of Early Prevention and Treatment of Regional High-Incidence-Tumors, Ministry of Education, Nanning 530021, PR China; 3 Guangxi Cancer Institute, Nanning 530021, PR China

## Abstract

To clarify the relationship between aldo-keto reductase family 1 member B10 (AKR1B10) expression and early hepatocellular carcinoma (HCC) recurrence, this study detected AKR1B10 expression in tumor and adjacent non-tumor tissues from 110 patients with hepatitis B virus (HBV)-related HCC underwent liver resection and analyzed its correlations with clinicopathological characteristics and prognosis of these patients. Detected by quantitative reverse transcription polymerase chain reaction, AKR1B10 mRNA expression showed significantly higher in HCC tissues than in adjacent non-tumor tissues, with a low level in normal liver tissues. Similar results was confirmed at the protein level using immunohistochemistry and Western blotting. High AKR1B10 expression was negatively correlated with serum alpha-fetoprotein level and positively correlated with HBV-DNA level. Patients with high AKR1B10 expression had significantly higher disease-free survival than those with low expression within 2 years after liver resection. Multivariate analysis also confirmed high AKR1B10 expression to be a predictor of low risk of early HCC recurrence. In addition, high AKR1B10 expression was found to be a favorable factor of overall survival. These results suggest that AKR1B10 is involved in HBV-related hepatocarcinogenesis, but its high expression could predict low risk of early tumor recurrence in patients with HBV-related HCC after liver resection.

Hepatocellular carcinoma (HCC) is the second most common cause of cancer death globally, responsible for 745,500 deaths and 782,500 new cases in 2012 alone^1^. China is a high-risk region of HCC, accounting for about half the worldwide number of HCC deaths and new HCC cases^1^,^2^. In 2015, up to 466,100 new cases occurred in the country, with the number set to increase because of population growth and aging^2^. Liver resection is considered as a curative treatment for HCC, however, tumor recurrence occurs in many patients after liver resection, leading their poor survival^3^,^4^. Recurrence rates at 5 years after liver resection exceeds 70%, and most recurrence occurs within 2 years^3^,^5^. Therefore, identifying biomarkers that predict early HCC recurrence can improve their management and help prolong their survival.

Aldo-keto reductase family 1 member B10 (AKR1B10) is a member of the aldo-keto reductase protein superfamily, a group of NAD(P)H dependent oxidoreductases implicated in xenobiotic detoxification, carcinogenesis, and cancer therapeutics^6^,^7^,^8^,^9^. AKR1B10 is expressed primarily in normal colon and small intestine, and at lower levels in normal liver^10^,^11^. Increasing evidence suggests that AKR1B10 plays an important role in carcinogenesis. Detoxification by AKR1B10 of reactive carbonyls and its regulation of fatty acid synthesis can promote the growth and proliferation of cancer cells^12^,^13^. AKR1B10 has been found to be overexpressed in smokers’ non-small cell lung carcinoma^14^, breast cancer^15^, pancreatic carcinoma^16^,^17^, and HCC^18^,^19^. Using random gene fishing, Heringlake *et al*.^18^ identified AKR1B10 as an overexpressed gene in HCC, and our laboratory has associated HCC with elevated AKR1B10 protein using quantitative proteomics^19^. These findings indicate that AKR1B10 may be involved in hepatocarcinogenesis.

Up to now, few studies focused on the impact of AKR1B10 on early HCC recurrence. Further more, the relationship between AKR1B10 expression and the prognosis of patients with HCC remains controversial. Ha *et al*.^20^ reported that high expression of AKR1B10 protein was a favorable factor for the prognosis of patients with HCC. On the contrary, Jin *et al*.^21^ associated high expression of AKR1B10 mRNA with poor disease-free survival (DFS) and overall survival (OS) in patients with HCC. These two studies also reported substantially different patterns of AKR1B10 expression in patients with HCC in different stages. Given these discrepancies and the fact that the patient populations in both studies were heterogeneous in terms of HCC risk factors, the possible relationship between AKR1B10 expression and early HCC recurrence remains unclear.

In China, more than 80% of patients with HCC have chronic hepatitis B virus (HBV) infection, which is the main risk factor for HCC development in this region^3^,^4^,^22^. To help elucidate the possible relationship between AKR1B10 expression and early HCC recurrence in a more homogeneous population, we examined a population of Chinese patients with HCC involving chronic HBV infection. This study aimed to clarify the relationship between AKR1B10 expression and early tumor recurrence in patients with HBV-related HCC after liver resection. The impact of AKR1B10 expression on HBV-related hepatocarcinogenesis was also evaluated.

## Results

During the study interval, a total of 258 patients underwent curative liver resection for HCC. Of these, 148 patients (57.4%) were excluded due to the following reasons: 41 patients (15.9%) had not HBV infection; 5 patients (1.9%) infected by hepatitis C virus (HCV); 64 patients (24.8%) received other anti-tumor treatments before liver resection; and 38 patients (14.7%) were lack of fresh tissue samples. The remaining 110 patients were included the study. In addition, another 8 samples of normal liver tissue were collected as controls.

### Patient characteristics

Clinicopathological characteristics of the 110 patients with HBV-related HCC are presented in Table 1. The patient population consisted of 96 males and 14 females, with a median age of 44 years (range, 25 to 71). Most patients (83.8%) had liver cirrhosis, and all had Child-Pugh A liver function. HCC stage according to the BCLC staging system was A in 60.9% of patients, B in 21.8% and C in 17.3%.

### Up-regulated expression of AKR1B10 in HBV-related HCC tissues

**Figure 1:**
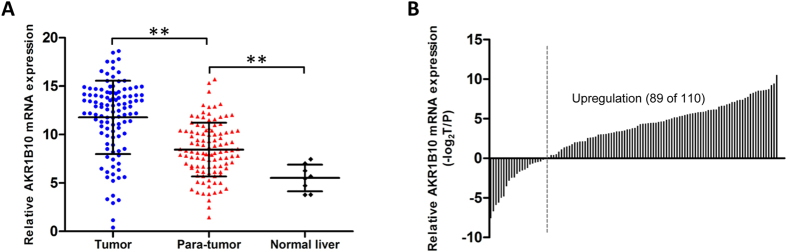
Up-regulated expression of AKR1B10 mRNA in hepatitis B virus (HBV)-related hepatocellular carcinoma (HCC) tissues. **(A)** Mean AKR1B10 mRNA level detected by qRT-PCR was significantly higher in HBV-related HCC tissues than in adjacent non-tumor tissues and significantly lower in normal liver tissues than in the adjacent non-tumor tissues. ^**^P < 0.01. **(B**) Relative levels of AKR1B10 mRNA were higher in HBV-related HCC tissues than in paired adjacent non-tumor tissues in 80.9% (89 of 110) of patients.

Levels of AKR1B10 mRNA were measured in 110 pairs of HBV-related HCC and adjacent non-tumor tissues as well as in 8 samples of normal liver tissue. Mean AKR1B10 mRNA level by quantitative reverse transcription polymerase chain reaction (qRT-PCR) was 11.77 ± 3.79 in HCC tissues, significantly higher than the level of 8.45 ± 2.78 in adjacent non-tumor tissues (*P* < 0.001; Fig. 1A). Relative levels of AKR1B10 mRNA were higher in HCC tissues than in paired adjacent non-tumor tissues in 80.9% of patients (Fig. 1B). In addition, levels of AKR1B10 mRNA were significantly lower in normal liver tissues than in the adjacent non-tumor tissues (5.52 ± 1.37 *vs* 8.45 ± 2.78, *P* = 0.004; Fig. 1A).

**Figure 2:**
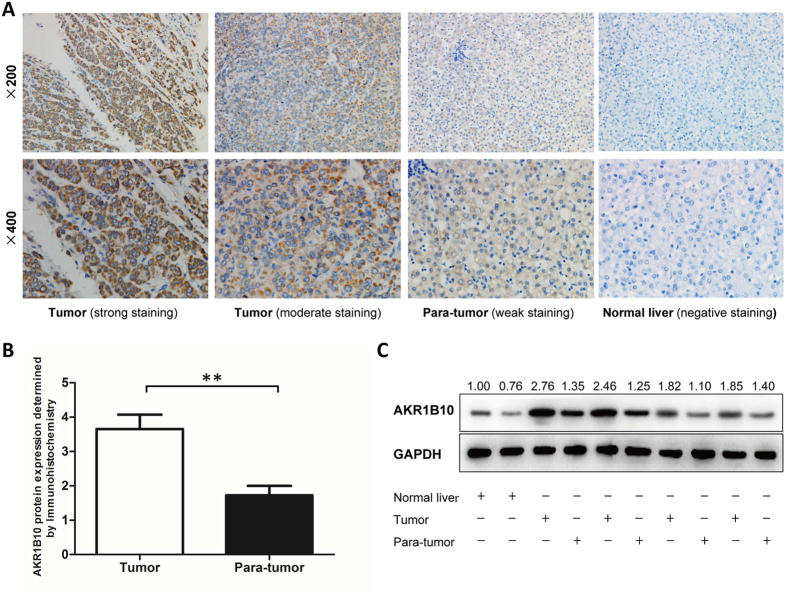
Up-regulated expression of AKR1B10 protein in hepatitis B virus (HBV)-related hepatocellular carcinoma (HCC) tissues. **(A)** Representative images of immunohistochemistry staining of AKR1B10 in HBV-related HCC, adjacent non-tumor, and normal liver tissues. **(B)** Mean AKR1B10 protein level detected by immunohistochemistry was significantly higher in HBV-related HCC tissues than in adjacent non-tumor tissues. ^**^P < 0.01. **(C**) AKR1B10 protein expression detected by Western blotting assay in 4 pairs of HBV-related HCC and adjacent non-tumor tissues as well as in 2 cases of normal liver tissue. (full-length blots are presented in Supplementary Figure 1 A and B; 1 A, AKR1B10; 1B, GAPDH).

Paraffin-embedded tissue blocks were available for 96 of the 110 patients, so AKR1B10 protein expression was semi-quantitated by immunohistochemistry in 96 pairs of HBV-related HCC and adjacent non-tumor tissues as well as in 8 cases of normal liver tissue. The proportion of samples positive for AKR1B10 was significantly higher for HCC tissues (61.5%) than for adjacent non-tumor tissues (37.5%, *P* = 0.001), and expression level was significantly higher in HCC tissues (*P* < 0.001, Fig. 2A and B). None of the normal liver tissues was positive for AKR1B10.

To confirm this finding, AKR1B10 protein expression was quantitatively detected by Western blotting assay in 4 pairs of HBV-related HCC and adjacent non-tumor tissues as well as in 2 cases of normal liver tissue. All the HCC tissues had higher level of AKR1B10 protein than their paired adjacent non-tumor tissues and both the two normal liver tissues showed low level of AKR1B10 protein. (Fig. 2C).

### Correlations between AKR1B10 expression and clinicopathological characteristics

Patients were stratified into groups showing high AKR1B10 mRNA expression (n = 67) and low expression (n = 43) based on the mean expression in the entire population. Then the two groups were compared in terms of clinicopathological characteristics (Table 1). The rate of high AKR1B10 expression was significantly higher among patients with serum alpha-fetoprotein (AFP) <200 ng/ml (32 of 44, 72.7%) than among those with serum AFP ≥200 ng/ml (35 of 66, 53.0%; *P* = 0.038). Besides, the rate of high AKR1B10 expression was significantly higher among patients with HBV-DNA ≥1000 copies/ml than among those with HBV-DNA <1000 copies/ml (*P* = 0.044). No obvious correlation was observed between AKR1B10 mRNA expression and gender, age, cirrhosis, tumor size or number, presence of macrovascular invasion, tumor stage, or degree of tumor cell differentiation.

### Correlation between AKR1B10 expression and early HCC recurrence

**Figure 3:**
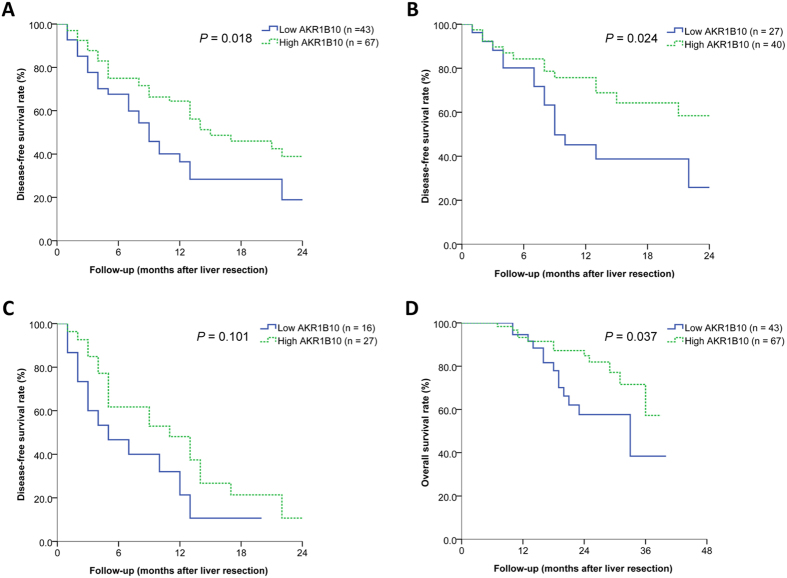
Correlations of AKR1B10 expression with early tumor recurrence and overall survival in patients with hepatitis B virus (HBV)-related hepatocellular carcinoma (HCC). (**A**) Among the total study population (n = 110), patients with high AKR1B10 expression had significantly higher disease-free survival (DFS) than those with low expression within 2 years after liver resection. (**B**) Among patients with BCLC stage A HCC (n = 67), DFS (within 2 years) was significantly higher among those with high AKR1B10 expression than among those with low expression. (**C**) Among patients with BCLC stage B or C HCC (n = 43), DFS (within 2 years) was not significantly higher among those with high AKR1B10 expression than among those with low expression. (**D**) Among the total study population, patients with high AKR1B10 expression had significantly higher overall survival than those with low expression.

A total of 60 (54.5%) patients experienced tumor recurrence within 2 years after liver resection, including 32 (47.8%) in the high AKR1B10 expression group and 28 (65.1%) in the low AKR1B10 expression group. Among all patients, median DFS was 13 months, and DFS rates were 72.2% at 6 months, 53.7% at 12 months, and 31.1% at 24 months. Patients with high AKR1B10 expression showed significantly higher DFS rates than those with low expression at 6 months (75.0 *vs* 67.6%), 12 months (64.4 *vs* 36.4%), and 24 months (38.9 *vs* 18.9%) (*P* = 0.018; Fig. 3A). Median DFS was 15 months in the high AKR1B10 expression group and 9 months in the low expression group.

Univariate and multivariate analyses confirmed high AKR1B10 expression to be an independent factor influencing early HCC recurrence (hazard ratio (HR) 0.572, 95% confidence interval (*CI*) 0.340–0.963, *P* = 0.036). Additionally, tumor size >5 cm, tumor number ≥2, presence of macrovascular invasion, and poor degree of tumor cell differentiation were also identified as independent factors influencing early HCC recurrence (Table 2).

Patients were further stratified by BCLC stage. Among patients with BCLC stage A HCC, DFS rates were significantly higher among those with high AKR1B10 expression than among those with low expression (*P* = 0.024; Fig. 3B). Among patients with BCLC stage B or C disease, DFS rates still tended to be higher among those with high AKR1B10 expression but the difference did not achieve significance (*P* = 0.101; Fig. 3C).

### Correlation between AKR1B10 expression and OS

Among all patients, 12 patients (17.9%) died in the high AKR1B10 expression group and 13 (30.2%) in the low AKR1B10 expression group during a mean (SD) follow-up of 20 (10) months. OS rates at 1, 2, and 3 years were significantly higher in the high AKR1B10 expression group (93.3%, 84.8%, and 57.3%) than in the low AKR1B10 expression group (94.6%, 57.6%, and 38.4%) (*P* = 0.040; Fig. 3D).

Univariate and multivariate analyses confirmed high AKR1B10 expression to be an independent predictor of OS (HR 0.429, 95%*CI* 0.193–0.951, *P* = 0.037). Presence of macrovascular invasion was also identified as a predictor of OS (Table 3).

## Discussion

AKR1B10 has been found to be up-regulated in various cancers and is considered as a potential tumor marker for smokers’ non-small cell lung carcinoma and breast cancer^14^,^15^. Our laboratory also identified AKR1B10 as an up-regulated protein in HCC using quantitative proteomics^19^. But the relationship between AKR1B10 expression and early tumor recurrence in patients with HCC is uncertain. In the present study, the expression of AKR1B10 was confirmed to be up-regulated in HBV-related HCC. Nevertheless, survival analysis showed that high expression of AKR1B10 was a predictor for low risk of early tumor recurrence in patients with HBV-related HCC after liver resection.

Using qRT-PCR, we found levels of AKR1B10 mRNA to be significantly higher in HBV-related HCC tissues than in adjacent non-tumor tissues, and we found that levels were higher in the adjacent non-tumor tissues from HCC patients than in normal liver tissues from patients with hepatic hemangioma without HBV or HCV infection. AKR1B10 up-regulation in HBV-related HCC tissues was confirmed at the protein level using immunohistochemistry and Western blotting. These findings indicate that high AKR1B10 expression is associated with HBV-related hepatocarcinogenesis. Consistent with this, a study of 119 chronic HBV-infected patients from Japan reported that high AKR1B10 expression in liver increased the risk of HCC development in these patients^23^. In addition, AKR1B10 expression correlated with serum levels of HBV-DNA in the present study. Patients with HBV-DNA ≥1000 copies/ml showed higher AKR1B10 expression than those with HBV-DNA <1000 copies/ml. These findings are consistent with the idea that HBV infection up-regulates AKR1B10 expression, which in turn promotes HBV-related hepatocarcinogenesis. Prospective studies are needed to test this hypothesis. This has important implications for timely diagnosis and treatment of HCC, since chronic HBV infection is one of its main causes, especially in China^4^,^24^.

AKR1B10 may promote carcinogenesis through various mechanisms. As an NAD(P)H dependent oxidoreductase, AKR1B10 catalyzes the reduction of aldehydes and ketones with broad substrate specificity^6^,^25^. For example, AKR1B10 converts retinals to retinols, which can deprive retinoic acid receptors of their ligands and reduce retinoic acid synthesis^26^. Retinoic acid is a critical regulator of cell proliferation and differentiation^27^,^28^. AKR1B10 can also affect cell proliferation by regulating the synthesis of long chain fatty acids, an important component of cell membrane^29^. Further more, AKR1B10 can protect cells against apoptosis induced by carbonyl groups by efficiently reducing carbonyl groups to hydroxyl groups^12^. This was confirmed by a study of Matkowskyj *et al*.^30^, which found that silencing AKR1B10 expression can increase cell apoptosis in HCC cells.

AFP has been widely used for screening and diagnosing HCC, but its sensitivity and specificity are limited^24^. In the present study, 44 patients (40%) had serum AFP levels <200 ng/ml. Interestingly, 72.7% of them showed high AKR1B10 expression in their HCC tissues. Therefore, AKR1B10 shows potential as a biomarker for identifying HCC in patients with low serum AFP levels. In the present study, we failed to find the correlation of AKR1B10 expression with macrovascular invasion or tumor size, number or stage, which is consistent with the results of Heringlake *et al*.^18^ However, some studies reported different findings. A study of 168 HCC patients from Germany in which 22% had HBV infection and 26% had HCV infection found that AKR1B10 protein overexpression correlated with lower tumor stage^31^. Conversely, a study of HCC patients, of whom 82% had HBV infection, found that AKR1B10 mRNA overexpression correlated with higher tumor stage^21^. These discrepancies may be caused by differences in HCC etiology or analysis methods.

Although AKR1B10 expression was found to be up-regulated in HBV-related HCC in this study, paradoxically, patients with high AKR1B10 expression had higher DFS within 2 years after liver resection for HCC. Multivariate analysis also identified high AKR1B10 expression as an independent predictor for low risk of early tumor recurrence in patients with HBV-related HCC after liver resection. Two studies in Korea^20^ and Taiwan^32^ reported results similar to ours: HCC patients with high AKR1B10 expression had better DFS than those with low AKR1B10 expression. However, contrary to our results, Jin *et al*.^21^ reported that HCC patients with high AKR1B10 expression had shorter DFS. It is possible that this result was confounded by the fact that their patients with high AKR1B10 expression also had higher tumor stage and higher rate of lymph node metastasis than patients with low AKR1B10 expression.

The apparently opposite effects of AKR1B10 expression on HBV-related HCC carcinogenesis and early recurrence may relate to recent findings that AKR1B10 silencing suppresses HCC cell proliferation, while AKR1B10 overexpression suppresses HCC cell invasion and migration^32^. These results are consistent with the idea that AKR1B10 promotes hepatocarcinogenesis but suppresses HCC metastasis. Indeed, work by Ha *et al*.^20^ and Liu *et al*.^32^ shows that high AKR1B10 expression correlates with low metastasis rate. These complex effects of AKR1B10 on HCC may reflect its ability to be regulated by numerous proteins: the promoter of the AKR1B10 gene contains binding sites for several oncogenic and tumor suppressor proteins, including c-Ets-1, C/EBP, AP-1, and p53^33^. Our results highlight the need to clarify the complex pathways that may link AKR1B10 with HCC onset and recurrence.

By stratified analyses, we further found that the expression of AKR1B10 significantly influenced early tumor recurrence in our patients with BCLC stage A HCC, but not in our patients with stage B or C disease. An possible explanation for this result is that tumor stage is a much stronger prognostic factor than AKR1B10 expression. Our results also suggest high AKR1B10 expression is a favorable factor for the OS of patients with HBV-related HCC, which is consistent with the result that high AKR1B10 expression negatively correlated with their early tumor recurrence.

The present study has several limitations. First, although this study revealed that AKR1B10 can promote hepatocarcinogenesis and decrease early HCC recurrence after liver resection, the impacts of AKR1B10 on the growth, invasion, and migration of HCC cells should be further explored. Second, follow-up in this study was insufficient to examine the relationship between AKR1B10 expression and long-term survival of patients with HBV-related HCC. Third, our institute is a referral center. Consequently, nearly 40% of the included patients were with intermediate or advanced HCC. The limitation of the source of cases may lead to selection bias. In addition, this is a relatively small study from a single medical center. Lack of external validation, our results may not extrapolate to all HBV-related HCC patients. Therefore, larger and multi-center studies are needed to confirm our results.

In conclusion, the present study suggests that AKR1B10 is involved in HBV-related hepatocarcinogenesis and may be a biomarker of HCC. AKR1B10 expression could be a useful predictor for early tumor recurrence in patients with HBV-related HCC after liver resection. High AKR1B10 expression could predict low risk of early tumor recurrence. Therefore, we suggest active adjuvant therapy and more frequent follow-up after liver resection in HCC patients with low AKR1B10 expression in order to improve prognosis.

## Methods

### Patients and tissue samples

From January 2013 to June 2014, all consecutive patients who underwent curative liver resection for HCC at the Affiliated Tumor Hospital of Guangxi Medical University were considered for this study. Curative liver resection was defined as complete resection of visible tumor and absence of cancer at surgical margins based on histological examination. Tumor stage was determined according to the Barcelona Clinical Liver Cancer staging system^24^. The inclusion criteria were: histologically confirmed HCC; a combination of chronic HBV infection; no infection of HCV; no treatment for HCC before liver resection; and availability of tissue samples. Fresh HCC tissues and paired adjacent non-tumor tissues of the included patients were obtained from the Tumor Tissue Bank of the Affiliated Tumor Hospital of Guangxi Medical University. As controls, samples of normal liver tissue were obtained from patients with hepatic hemangioma (confirmed by histological examination) without HBV or HCV infection who underwent liver resection. Ethical approval was obtained from the Ethics Committee of the Affiliated Tumor Hospital of Guangxi Medical University, and all experiments were performed according to the Declaration of Helsinki 2013 edition. Written informed consent was acquired from each enrolled patient.

### Follow-up

After liver resection, all patients were followed up periodically. Serum assay of AFP, chest X-ray, abdominal computed tomography (CT) or magnetic resonance imaging (MRI), and abdominal ultrasonography were conducted at 1 month after liver resection and every 3 months thereafter. The last follow-up was conducted in June 2016. Early HCC recurrence was defined as tumor recurrence within 2 years after liver resection^5^. HCC recurrence was treated as described in our previous study^34^.

### qRT-PCR

Total RNA was extracted from tissues using Trizol reagent (Invitrogen, Grand Island, NY, USA). Complementary DNA (cDNA) was generated using PrimeScript™ reverse transcription reagent Kit (TAKARA, Japan) according to the manufacturer’s instructions. PCR amplification was performed using the SYBR^®^ Premix Ex Taq™ Kit (TAKARA, Japan) according to the manufacturer’s instructions, and reactions were analyzed using the ABI StepOne Plus System (Applied Biosystems, Foster City, CA, USA). Primers to amplify the AKR1B10 gene were 5′-CCCAAAGATGATAAAGGTAATGCCATCGGT-3′ (forward) and 5′-CGATCTGGAAGTGGCTGAAATTGGAGA-3′ (reverse). Expression of the AKR1B10 gene was normalized to that of glyceraldehyde 3-phosphate dehydrogenase (GAPDH), which was amplified using the primers 5′-ATGACCCCTTCATTGACC-3′(forward) and 5′-GAAGATGGTGATGGGATTTC-3′ (reverse). All primers were synthesized by Shanghai Biological Engineering Co. (Shanghai, China).

### Immunohistochemistry

Tissues for immunohistochemistry were fixed with 10% formalin, then embedded in paraffin. Sections (3 mm) from paraffin-embedded blocks were deparaffinized in xylene, then rehydrated in a descending ethanol gradient. Antigen retrieval was carried out by pressure cooking in Tris/EDTA (pH 9.0). Tissue sections were incubated with 3% hydrogen peroxide for 15 min to block the activity of endogenous peroxidase. After 3 washes with phosphate-buffered saline (PBS) for 3 min each. the sections were incubated at room temperature for 2 h with rabbit monoclonal anti-AKR1B10 antibody (1:800; Abcam, Cambridge, USA). After 3 washes with PBS, sections were incubated at room temperature for 20 min with goat anti-rabbit biotin-conjugated secondary antibody (ZSGB, Beijing, China), then stained with 3,3-diaminobenzidine tetrahydrochloride. AKR1B10 protein expression was semi-quantitated based on immunohistochemistry by scoring staining intensity on a 4-point scale (0, negative; 1, weak; 2, moderate; 3, strong) and scoring the proportion of positive cells on a 5-point scale (0, 0%; 1, 1–25%; 2, 26–50%; 3, 51–75%; 4, 76–100%). The product of the two scores determined the final staining result.

### Western blotting assay

Tissues were lysed in RIPA buffer (Solarbio, Beijing, China) containing 1 mmol/l PMSF (Solarbio, Beijing, China), and proteins were collected by centrifugation at 12000 *g* at 4 °C for 10 min. Protein concentrations were determined using the BCA protein assay kit (Beyotime, Shanghai, China). Equal amounts of protein (20 μg) were separated by 10% sodium dodecyl sulfate-polyacrylamide gel electrophoresis and then transferred to polyvinylidene difluoridemembranes (Millipore, MA, USA). Membranes were blocked for 2 h with 5% skim milk in PBS containing Tween-20 (PBST), and then incubated overnight at 4 °C with rabbit monoclonal anti-AKR1B10 antibody (1:1000; Abcam, Cambridge, USA). GAPDH was also detected to serve as an internal control. After 3 washes in PBST for 5 min each, membranes were incubated for 2 h at room temperature with goat anti-rabbit horseradish peroxidase-conjugated antibody (1:5000; Abcam, Cambridge, USA). Bands were visualized using an Enhanced Chemiluminescence kit (Beyotime, Shanghai, China). All experiments were carried out 3 times.

### Statistical analysis

To minimize the risk of bias, blinding of outcome assessors and data analysts was carried out during the processes of outcome assessment and data analysis. Statistical analyses were carried out with SPSS 19.0 (IBM, USA), and *P* < 0.05 was considered significant. Continuous data were expressed as mean ± SD and compared between groups using Student’s *t* test. Categorical data were analyzed using 2-sided chi-square test or Fisher’s exact test, as appropriate. The Kaplan–Meier method was used to calculate DFS and OS, and differences in survival curves between groups were compared using the log-rank test. Independent predictors of early tumor recurrence or OS in patients with HCC after liver resection were explored using multivariate analysis based on Cox proportional hazards regression model.

## Additional Information

**How to cite this article**: Wang, Y.-Y. *et al*. High expression of AKR1B10 predicts low risk of early tumor recurrence in patients with hepatitis B virus-related hepatocellular carcinoma. *Sci. Rep.* **7**, 42199; doi: 10.1038/srep42199 (2017).

**Publisher's note:** Springer Nature remains neutral with regard to jurisdictional claims in published maps and institutional affiliations.

## Supplementary Material

Supplementary Figure 1

## Figures and Tables

**Table 1:** Correlations between AKR1B10 expression and clinicopathologic characteristics of patients with HBV-related hepatocellular carcinoma.

Characteristics	n	AKR1B10 expression		*χ*^*2*^	*P* value
		Low n (%)	High n (%)		
Gender					
Male	96	37 (38.5)	59 (61.5)	0.096	0.757
Female	14	6 (42.9)	8 (57.1)		
Age (years)					
<60	86	33 (38.4)	53 (61.6)	0.086	0.770
≥60	24	10 (41.7)	14 (58.3)		
AFP (ng/ml)					
<200	44	12 (27.3)	32 (72.7)	4.302	0.038
≥200	66	31 (47.0)	35 (53.0)		
HBV-DNA (copies/ml)					
<1000	27	15 (55.6)	12 (44.4)	4.074	0.044
≥1000	83	28 (33.7)	55 (66.3)		
Cirrhosis					
Yes	92	34 (37.0)	58 (63.0)	1.076	0.300
No	18	9 (50.0)	9 (50.0)		
Tumor size (cm)					
≤5	37	11 (29.7)	26 (70.3)	2.052	0.152
>5	73	32 (43.8)	41 (56.2)		
Tumor number					
1	83	31 (37.3)	52 (62.7)	0.431	0.512
≥2	27	12 (44.4)	15 (55.6)		
Macrovascular invasion					
Yes	19	7 (36.8)	12 (63.2)	0.049	0.825
No	91	36 (39.6)	55 (60.4)		
BCLC stage					
A	67	27 (40.3)	40 (59.7)	0.105	0.746
B or C	43	16 (37.2)	27 (62.8)		
Differentiation degree					
Well or moderately	68	25 (46.8)	43 (63.2)	0.405	0.525
Poorly	42	18 (42.9)	24 (57.1)		

AFP, alpha-fetoprotein; BCLC, Barcelona Clinical Liver Cancer; hepatitis B virus.

**Table 2:** Univariate and multivariate analyses to identify factors influencing early tumor recurrence in patients with HBV-related hepatocellular carcinoma after liver resection.

	Univariate analysis		Multivariate analysis	
	Hazard ratio (95% *CI*)	*P*	Hazard ratio (95% *CI*)	*P*
Gender (female)	0.906 (0.389–2.110)	0.819	—	—
Age ≥60	0.916 (0.475–1.765)	0.793	—	—
AFP ≥200 ng/ml	0.984 (0.587–1.651)	0.952	—	—
HBV-DNA ≥1000 copies/ml	1.052 (0.568–1.948)	0.872	—	—
Cirrhosis	1.406 (0.667–2.965)	0.371	—	—
Tumor size >5 cm	2.396 (1.271–4.515)	0.007	2.513 (1.304–4.845)	0.006
Tumor number ≥2	1.468 (0.850–2.534)	0.068	1.921 (1.085–3.403)	0.025
Macrovascular invasion	2.680 (1.473–4.877)	0.001	2.824 (1.520–5.249)	0.001
Differentiation degree (poorly)	1.757 (1.057–2.924)	0.030	1.767 (1.054–2.967)	0.031
AKR1B10 expression (high)	0.555 (0.333–0.925)	0.024	0.572 (0.340–0.963)	0.036

Factors with a probability threshold of less than 0.100 in univariate analysis were selected into the multivariate Cox regression model. AFP, alpha-fetoprotein; *CI*, confidence interval; HBV, hepatitis B virus.

**Table 3:** Univariate and multivariate analyses to identify factors influencing overall survival in patients with HBV-related hepatocellular carcinoma after liver resection.

	Univariate analysis		Multivariate analysis	
	Hazard ratio (95% *CI*)	*P*	Hazard ratio (95% *CI*)	*P*
Gender (female)	0.783 (0.183–3.344)	0.741	—	—
Age ≥60	0.582 (0.174–1.949)	0.380	—	—
AFP ≥200 ng/ml	1.003 (0.454–2.216)	0.995	—	—
HBV-DNA ≥1000 copies/ml	0.581 (0.238–1.423)	0.235	—	—
Cirrhosis	2.283 (0.537–9.701)	0.263	—	—
Tumor size >5 cm	1.621 (0.645–4.076)	0.304	—	—
Tumor number ≥2	1.421 (0.610–3.309)	0.416	—	—
Macrovascular invasion	3.551 (1.579–7.985)	0.002	3.662 (1.630–8.230)	0.002
Differentiation degree (poorly)	1.647 (0.741–3.663)	0.221	—	—
AKR1B10 expression (high)	0.447 (0.202–0.988)	0.047	0.429 (0.193–0.951)	0.037

Factors with a probability threshold of less than 0.100 in univariate analysis were selected into the multivariate Cox regression model. AFP, alpha-fetoprotein; *CI*, confidence interval; HBV, hepatitis B virus.
