# Supplementary material for: High expression of AKR1B10 predicts low risk of early tumor recurrence in patients with hepatitis B virus-related hepatocellular carcinoma
Source: Sci Rep. 2017 Feb 9;7:42199. doi: 10.1038/srep42199 (PMC5299837; doi:10.1038/srep42199)

Supplementary Information

High expression of AKR1B10 predicts low risk of early tumor recurrence in patients with hepatitis B virus-related hepatocellular carcinoma

Yan-Yan Wang[1,3,*], Lu-Nan Qi[1,2,3,*], Jian-Hong Zhong[1,2,3], Hong-Gui Qin[1,3], Jia-Zhou Ye[1,2,3], Shi-Dong Lu[1,3], Liang Ma[1,2,3], Xue-Mei You[1,2,3], Bang-De Xiang[1,2,3], and Le-Qun Li[1,2,3]

1 Department of Hepatobiliary Surgery, Affiliated Tumor Hospital of Guangxi Medical University, Nanning 530021, PR China

2 Guangxi Cancer Institute, Nanning 530021, PR China

3 Key Laboratory of Early Prevention and Treatment of Regional High-Incidence-Tumors, Ministry of Education, Nanning 530021, PR China

*These authors contributed equally to this work.

Correspondence to: Dr Le-Qun Li (email: wyy857355056@163.com) or Xue-Mei You (email: 397845319@qq.com), Department of Hepatobiliary Surgery, Affiliated Tumor Hospital of Guangxi Medical University, He Di Rd. #71, Nanning 530021, China. Phone: +86-771-5330855. Fax: +86-771-5312000.

Manuscript type: original article.

SUPPLEMENTARY FIGURES

Supplementary Figure 1. Full-length blots of AKR1B10 (A) and GAPDH (B) detected by Western blotting assay.

A

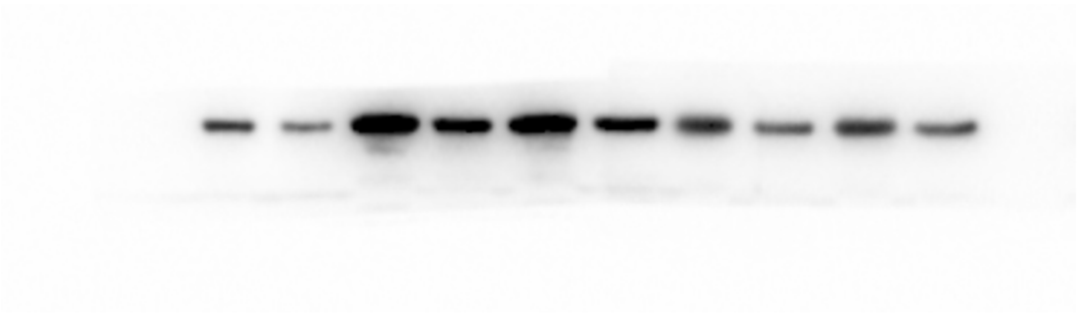

B

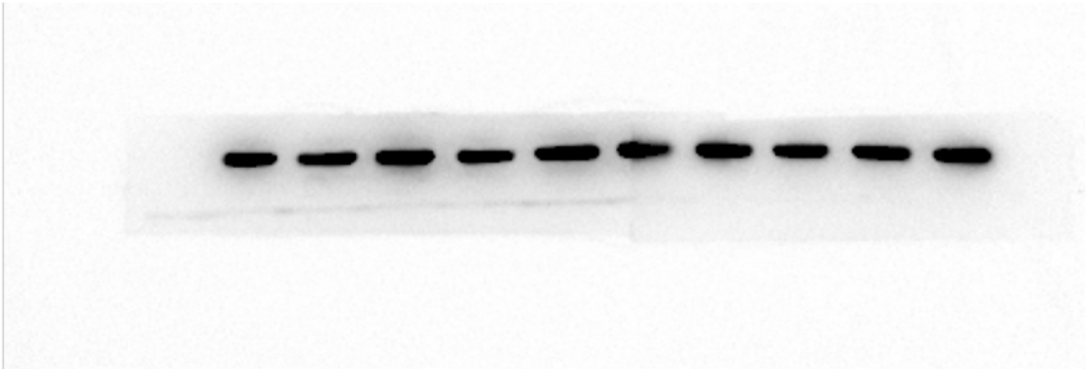

Supplement: Supplementary Figure 1 [file srep42199-s1.pdf]
